# A computational study on the influence of antegrade accessory pathway location on the 12-lead electrocardiogram in Wolff–Parkinson–White syndrome

**DOI:** 10.1093/europace/euae223

**Published:** 2024-09-11

**Authors:** Karli Gillette, Benjamin Winkler, Stefan Kurath-Koller, Daniel Scherr, Edward J Vigmond, Markus Bär, Gernot Plank

**Affiliations:** Division of Biophysics and Medical Physics, Gottfried Schatz Research Center, Medical University of Graz, Neue Stiftingtalstraße 6, 8010 Graz, Austria; BioTechMed-Graz, Mozartgasse 12/II, 8010 Graz, Austria; Physikalisch-Technische Bundesanstalt, National Metrology Institute, Berlin, Germany; Division of Pediatric Cardiology, Department of Pediatrics, Medical University of Graz, Graz, Austria; Department of Cardiology, Medical University of Graz, Graz, Austria; IHU Liryc, Electrophysiology and Heart Modeling Institute, Fondation University Bordeaux, Pessac-Bordeaux, France; Institute of Mathematics of Bordeaux, UMR 5251, University Bordeaux, Talence, France; Physikalisch-Technische Bundesanstalt, National Metrology Institute, Berlin, Germany; Division of Biophysics and Medical Physics, Gottfried Schatz Research Center, Medical University of Graz, Neue Stiftingtalstraße 6, 8010 Graz, Austria; BioTechMed-Graz, Mozartgasse 12/II, 8010 Graz, Austria

**Keywords:** Wolff–Parkinson–White syndrome, Cardiac digital twins, 12-lead ECG, Uncertainty quantification, Sensitivity analysis, Accessory pathways, Virtual models of cardiac electrophysiology

## Abstract

**Aims:**

Wolff–Parkinson–White (WPW) syndrome is a cardiovascular disease characterized by abnormal atrioventricular conduction facilitated by accessory pathways (APs). Invasive catheter ablation of the AP represents the primary treatment modality. Accurate localization of APs is crucial for successful ablation outcomes, but current diagnostic algorithms based on the 12-lead electrocardiogram (ECG) often struggle with precise determination of AP locations. In order to gain insight into the mechanisms underlying localization failures observed in current diagnostic algorithms, we employ a virtual cardiac model to elucidate the relationship between AP location and ECG morphology.

**Methods and results:**

We first introduce a cardiac model of electrophysiology that was specifically tailored to represent antegrade APs in the form of a short atrioventricular bypass tract. Locations of antegrade APs were then automatically swept across both ventricles in the virtual model to generate a synthetic ECG database consisting of 9271 signals. Regional grouping of antegrade APs revealed overarching morphological patterns originating from diverse cardiac regions. We then applied variance-based sensitivity analysis relying on polynomial chaos expansion on the ECG database to mathematically quantify how variation in AP location and timing relates to morphological variation in the 12-lead ECG. We utilized our mechanistic virtual model to showcase the limitations of AP localization using standard ECG-based algorithms and provide mechanistic explanations through exemplary simulations.

**Conclusion:**

Our findings highlight the potential of virtual models of cardiac electrophysiology not only to deepen our understanding of the underlying mechanisms of WPW syndrome but also to potentially enhance the diagnostic accuracy of ECG-based algorithms and facilitate personalized treatment planning.

**What’s new?**

- We present a physiologically detailed virtual model of cardiac electrophysiology that represents Wolff–Parkinson–White syndrome with an accessory pathway manifesting as an antegrade bypass tract.
- We quantified how sensitive the morphology of the 12-lead electrocardiogram (ECG) is to the location and timing of pre-ventricular ventricular excitation of an accessory pathway manifesting as an antegrade bypass tract using sensitivity analysis.
- We demonstrate failure mechanisms of ECG-based localization algorithms to accurately localize accessory pathways inserting on the right and left side of the septum using exemplary simulations.

## Introduction

Wolff–Parkinson–White (WPW) syndrome is a heart rhythm disorder that is characterized by the presence of at least one or more accessory pathways (APs) facilitating abnormal atrioventricular conduction. WPW typically manifests in children and affects between 0.03% and 0.5%in the general population.^1,2^ Without treatment, the condition can lead to paroxysmal palpitations and morbidity through supraventricular tachycardias or sudden cardiac arrest.^1,3^ Treatment of WPW syndrome typically involves invasive catheter ablation in efforts to restore the normal heart rhythm. Approximately, 6% of ablations are still unsuccessful.^4,2,5^

From a clinical perspective, accurate prediction of the location of the AP is thus of great value as it relates to procedural planning, risk prediction, and procedural outcome.^6^ Determining whether an antegrade AP exists in the right or left ventricles (RV or LV) guides the decision to perform trans-septal puncture or retro-aortic approach, respectively. Discrimination of left-sided APs also allows for pre-procedural informed consent emphasizing trans-septal/retro-aortic approach and associated risks (e.g. arterial injury) compared to strictly right-sided catheterization. Right-sided lateral pathways may implicate lower success of ablation due to frequently appearing arborization of APs.^7^ Right-sided anterolateral or anterior APs may represent as a duplication of the His–Punkinje system necessitating more extensive ablation and different tools (e.g. steerable sheaths) compared to ‘standard’ AP ablation procedures.^8^ Accurately predicting septal pathways is also of special interest to physicians because of the potential vicinity to the intrinsic conduction system, especially the His-bundle.^9^ Parahisian AP ablation procedures may require extensive mapping of atrial and ventricular insertion sites aiming to find a target site far enough distant to the His-bundle to safely perform ablation. Procedure time may increase significantly for these specific AP locations. The risk of injuring the intrinsic conduction system and subsequent pacemaker implantation is also higher in e.g. anteroseptal/parahisian APs compared to free wall APs.

Established clinical diagnostic trees for pre-procedural localization of the AP inferred from the 12-lead electrocardiogram (ECG) exist.^10–15^ These algorithms depend on identifying morphological features in the 12-lead ECG that uniquely indicate pre-excitation within anatomical sites across the atrioventricular junction region and thus maximize differentiation. Current ECG algorithms and diagnostic trees are primarily built upon data acquired through general clinical studies that aim to understand the influence of WPW manifestation on clinically relevant ECG metrics, typically within the delta wave and QRS complex.^16,17^ For example, both El Hamriti *et al.*^11^ and Pambrun *et al.*^12^ rely on the polarity of the V1 lead to differentiate between the mitral and tricuspid valves, respectively, relating to the LV and RV. The Easy-WPW algorithm by El Hamriti *et al.*^11^ then relies on what leads exhibit the most positive delta waves, and the QRS transition in the precordial leads. Pambrun *et al.*^12^ subsequently rely on the number of positive inferior leads (II, aVF, and III). Further differentiation in right-sided APs relies on the polarity of V3, while the V1/I ratio and lead II morphology are utilized within the LV.

Current ECG algorithms for AP localization prediction show acceptable accuracy for the determination of left- vs. right-sided APs, but still suffer difficulties predicting the exact location.^18^ Furthermore, such diagnostic trees may lack sensitivity and specificity within regions such as the septum, are not as accurate on a paediatric population,^11,12^ and fail under more complex manifestations of the disease, especially when compounded with multiple pathways or additional cardiac disorders.^19^ In situations where localization fails with a standard ablation catheter, an electrophysiological study with electro-anatomical mapping is performed to better elucidate AP location. Doing so results in increased procedural time and radiation exposure to both patient and personnel, as well as increased cost due to the use of a mapping catheter.

Virtual models of cardiac electrophysiology that are capable of representing WPW syndrome could be used to overcome such limitations. In general, virtual models of cardiac electrophysiology are becoming increasingly feasible for exploring the mechanisms behind various cardiac diseases, serving as clinical tools, and generating data to supplement clinical data for machine learning approaches.^20–22^ When the virtual models are personalized in terms of both anatomy and electrophysiology, often referred to as a cardiac digital twin or patient-specific model, they can be used for precision medicine in the form of prognostics, diagnostics, and treatment planning. While computational models have been used to study WPW,^23,24^ such studies have not been performed in a whole-heart personalized model capable of capturing the patient-specific electrophysiological detail of the disease.

Furthermore, sensitivity analysis (SA) methods on patient-specific models of WPW syndrome have not been yet deployed to understand how the location of the AP influences the morphology of the 12-lead ECG and, therefore, the success of localization algorithms. Performing sensitivity analysis and related uncertainty quantification on the virtual models provides a systematic framework to mathematically quantify how variation in the electrophysiological excitation (or a derived 12-lead ECG signal) is related to underlying model parameters. SA and uncertainty quantification have, thus, been recently deployed to the virtual models in various forms to different cardiac problems at different scales to better understand parameter-output relationships.^25–28^ Employing the variance-based SA method called polynomial chaos expansion (PCE)^29^ provides the global sensitivities for a given set of model evaluations. PCE has been recently applied to cardiac modelling,^30–34^ providing parameter sensitivities in the form of Sobol coefficients over the entire 12-lead ECG, enabling the localization of the signal change with respect to both timing and lead.

We utilized a virtual model of cardiac electrophysiology of WPW to provide insight into the genesis of morphological changes in 12-lead ECG that could influence pre-procedural localization. A whole-heart patient-specific model was constructed using an automated pipeline for a single male patient with normal sinus rhythm and adapted for WPW in previous work.^35,36^ A short atrioventricular bypass tract allowing antegrade conduction was inserted within the patient-specific model to allow abnormal conduction from the atria to the ventricles to facilitate pre-ventricular excitation. Various locations of antegrade APs were automatically swept using latin-hyper-cube sampling to generate a synthetic 12-lead ECG database. All ECGs within the database were grouped according to regions within the clinically standard bullseye plot to observe regional variation in 12-lead ECG morphology. How strongly AP location influences morphology in the 12-lead ECG was quantified by SA utilizing a PCE framework. Exemplary simulations of APs located on either side of the septum were conducted to showcase possible mechanisms behind localization failure of common ECG algorithms. All results are discussed in the context of ECG criteria as determined by clinically utilized algorithms of Pambrun *et al.*^12^ and El Hamriti *et al*.^11^

## Methods

**Figure 1.**
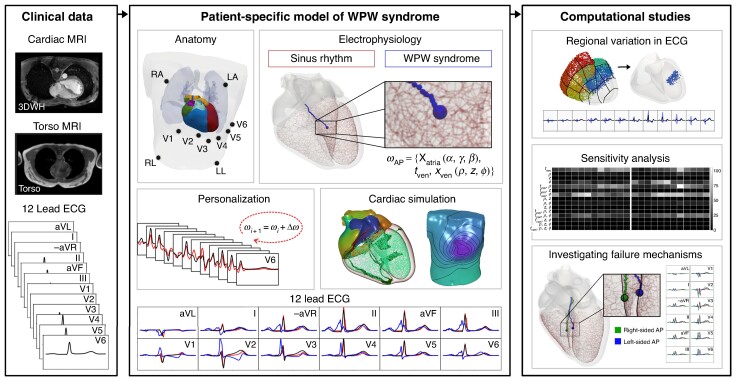
We develop a patient-specific model of Wolff–Parkinson–White (WPW) syndrome from clinical magnetic resonances images that has been personalized with the 12-lead electrocardiogram (ECG) under sinus rhythm. Using efficient cardiac simulation, we perform three computational studies. AP, accessory pathway; MRI, magnetic resonances image.

A model of WPW assuming antegrade AP conduction was first generated within a pre-existing patient-specific model for a single male subject. A synthetic ECG database was then generated by automatically varying the AP location. The database was generated using a modelling pipeline constructed within the openCARP framework.^37^ To quantify how strongly AP location influenced variation in signal morphology within the 12-lead ECG, the 12-lead ECGs were regionally grouped according to a bullseye plot and UQ with PCE was applied. A graphical abstract provides the methodological overview (*Figure* 1).

### Acquisition of clinical data

Clinical data were acquired for a single male patient of 45 years under normal sinus rhythm. Two magnetic resonance images (MRIs) were acquired sequentially: (i) a 3D whole-heart cardiac MRI with isotropic resolution 0.7×0.7×  $0.7{m\;m}^{3}$ and (ii) a 4-stack torso MRI with resolution 1.3×1.3×  $3.0{\mathrm{mm}}^{3}$. Both scans were taken at 3T (Magnetom Skyra, Siemens Healthcare, Erlangen, Germany). Before MRI acquisition, the 12-lead ECG was acquired using MRI-compatible electrodes that were left intact during acquisition. The clinical 12-lead ECG had been filtered with a 150 Hz low pass filter, a 50 Hz bandstop filter, and a high pass filter of 0.05 Hz. The MRI study was approved by the ethical review board of the Medical University of Graz (EKNr: 24–126 ex 11/12). The subject gave written informed consent.

### Construction of the patient-specific model

To generate a patient-specific anatomical model, automatic segmentation of the cardiac MRI was performed using a convolutional neural network.^38^ The heart segmentation was corrected manually in open-source *Seg3D* with the assistance of algorithms predominately based on intensity thresholds.^39^ This included removing disconnected tissue, closing any holes, and making sure all surfaces intersected properly. The torso and lungs were also segmented from the torso MRI stack using semi-automatic approaches based on thresholds and registered with the heart using a built-in iterative closest point algorithm within Seg3D (see Tools/Point Set Registration).^40^ From the joint segmentation, a finite-element volumetric mesh with tetrahedral elements of approximately 900μm resolution within the heart and up to 4500μm in the torso and lungs was generated using Tarantula^41^(CAE Software Solutions, Eggenburg, Austria). The resolution is reported as average edge length across the mesh. The model was subsequently equipped with rule-based myocardial fibres generated in both the ventricles^42^ and the atria,^43^ separately. To facilitate automated control and variation of electrophysiological parameters, such as the location of the AP, universal cardiac coordinates were generated for the ventricles^44,45^ and the atria.^46^ An electrical isolation layer between the atria and the ventricles was enforced using nodal splitting. The RV outflow tract was assumed to be electrically inert. Electrode locations of the 12-lead ECG were recovered on the torso surface from the location of the MRI-compatible electrodes.

Aspects of the cardiac electrophysiology of the patient-specific model were subsequently personalized to generate a 12-lead ECG replicating the measured beat-averaged 12-lead sinus rhythm ECG.^36^ Within the atria, the anatomical features of the sino-atrial node, Bachmann’s bundle, and the foramen ovale within the atria were placed to facilitate faster inter-atrial conduction per the measured P-wave duration and morphology.^36^ Generic parameters were used for atrial electrophysiology, and no calibration was performed. The timings and locations of the primary root sites of the five fascicles of the His–Purkinje system^47,48^ facilitating ventricular activation were personalized using a refined sampling procedure.^45^ The optimized sites and timings were then replaced with a physiological His–Purkinje System.^47^ Repolarization gradients were prescribed using a linear mapping between activation and action potential duration as detailed in^36^ leading to a realistic T-wave. The personalized signal resulted in an average L2-norm difference of 0.08 under normal sinus rhythm as reported in previous work.^36^ Full details of the model and other electrophysiological parameter settings are available in.^45,47,36^

### Representation of the antegrade accessory pathway

A short atrioventricular bypass tract allowing antegrade conduction was added into the patient-specific model to replicate ventricular pre-excitation caused by WPW. Corresponding insertion and exit points of the AP were defined using anatomically intuitive universal cardiac coordinates, respectively. Exact localization of the sites into nodes existing on the mesh was facilitated through open-source *meshtool* that relies on a custom-built K-Dimensional tree algorithm.^49^ A geodesic path was then found between each corresponding insertion and exit site to determine the length of the pathway *d*. The cable of the pathway was prescribed a given conduction velocity ${\mathrm{CV}}_{\mathrm{AP}}$. Given this representation, a parameter vector $\omega_{\mathrm{AP}}$ consisting of a total of seven parameters can be used to define the antegrade AP leading to pre-excitation within the ventricles:


(1) $$\omega_{\mathrm{AP}}=\{x_{\mathrm{atria}}(\alpha ,\gamma ,\beta ),{\mathrm{CV}}_{\mathrm{AP}},x_{\mathrm{ven}}(\rho ,z,\phi )\},$$

where $x_{\mathrm{atria}}$ and $x_{\mathrm{ven}}$ are defined with universal cardiac coordinates and ${\mathrm{CV}}_{\mathrm{AP}}$ is the conduction velocity of the AP. The sites $x_{\mathrm{ven}}$ and $x_{\mathrm{atira}}$ were localized to the closest node in the mesh using *meshtool*. The prescribed timing of the pre-excitation in the ventricles ($t_{\mathrm{ven}}$) could, therefore, be computed from the activation time at the atrial insertion site plus the time required to traverse the pathway.


$$t_{\mathrm{ven}}=\frac{d}{CV_{\mathrm{AP}}}+t_{\mathrm{atria}},$$

where $t_{\mathrm{atria}}$ is the time at which the excitation wavefront reaches $x_{\mathrm{atria}}$. For full details on the construction of the pathways, please refer to Supplementary material online, *S1*.

### Sampling schematic

A synthetic ECG database was generated by varying AP locations across both ventricles. All APs were assumed to propagate activation from the atria to the ventricles leading to ventricular pre-excitation. The RV outflow tract was taken to be inert and thus APs were neglected within this area. Latin hypercube sampling was used to generate the sampling schematic for $x_{\mathrm{atria}}$ and $x_{\mathrm{ven}}$ defined in Eq. (1). The maximal allowed distance apart was 6cm to ensure a uniform sampling of $x_{\mathrm{ven}}$ across the top of the ventricles in the upper two apicobasal regions in the bullseye plot. A conduction velocity in the accessory pathway (${\mathrm{CV}}_{\mathrm{AP}}$) of $2.0{m\;s}^{-}1$ was set to replicate Purkinje fibres in agreement with electrophysiological studies.^50^ A final total of 4678 APs in the LV and 4593 in the RV were thus actually sampled across the atrioventricular junction region. The database was split into LV and RV datasets for subsequent processing. For full details on the sampling schematic, please refer to the Supplementary material online, *S2*.

### Cardiac simulation

All electrophysiological simulations during both normal sinus rhythm and in the presence of AP were run with CARPentry^51^ using 16 cores on a desktop machine. All simulations were run for 700m s. Cardiac sources were represented using the reaction-Eikonal method in monodomain mode without diffusion to recover membrane voltages within the heart.^52^ Note that use of an Eikonal-based approach allows for simulating pre-ventricular activation from a single node in the mesh, i.e. for $x_{\mathrm{ven}}$, without a source-sink mismatch that could occur in traditional reaction-diffusion approaches.

Electrical potentials at electrodes with known positions on the torso surface from the MRI were acquired using lead field projection of the simulated membrane voltages.^53^ The 12-lead ECG was then constructed using the traditional combination of the electrical potentials.^54^ The cardiac simulation framework is described in detail in Gillette *et al.*^45^ and utilized previously in Gillette *et al.*^35^ All simulated 12-lead ECGs were filtered with a 150 Hz low pass filter according to clinical settings used during clinical ECG acquisition. A global scaling factor of 0.32 was previously computed to minimize the L2 norm between the synthetic personalized 12-lead ECG and the measured one during normal sinus rhythm in previous work^36^ and was applied to all simulated 12-lead ECGs.

### Regional separation of the 12-lead ECGs

**Figure 2.**
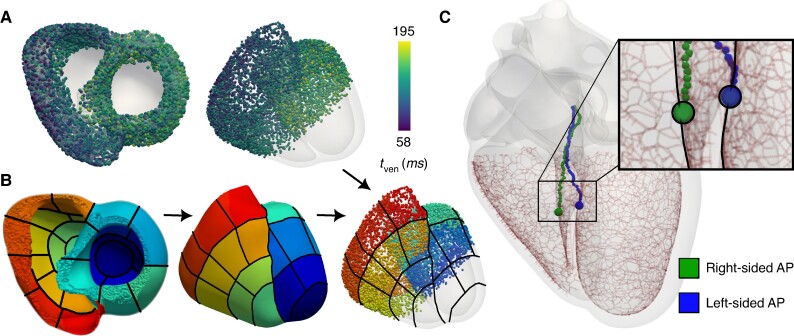
(*A*) Sampling sites were selected across the upper sections of the ventricles within the timings used to construct the 12-lead ECG Database. (*B*) Sampled sites are organized into segments on the bullseye plot. (*C*) The two septal APs corresponding to the exemplary simulations are located on either side of the septum, but in the area of the His–Purkinje System. AP, accessory pathway; ECG, electrocardiogram.

The sources simulated 12-lead ECGs were regionally grouped according to a standard 17-segment bullseye plot within the LV that was extrapolated to the RV (*Figure* 2*B*). For exact details on the construction of the bullseye plot using universal cardiac coordinates, we refer to Supplementary material online, *S3*. Within each region, the mean signal and the mean signal plus or minus two standard deviations were computed for all 12-lead ECGs within that particular region to demonstrate overall signal variation.

### Sensitivity analysis

Quantifying the sensitivity of the 12-lead ECGs from the database with respect to the four different input parameters of *ρ*, *z*, *ϕ*, and $t_{\mathrm{ven}}$ in $\omega_{\mathrm{AP}}$ was performed by employing PCE^29^ in each ventricle separately. We applied the Python toolbox ‘PyThia UQ’ that determines PCE expansion coefficients via a multi-linear least-squares regression.^55^

To compute the time-dependent sensitivity $\hat{S}(t)$ of each parameter in $\omega_{\mathrm{AP}}$, we multiply the obtained Sobol indices $S(t{)}_{i}$—where *i* stands for a multi-index numbering all possible parameter combinations—with the standard deviation of the signal σ(t) at each point in time:


(2) $$\hat{S}(t{)}_{i}=S_{i}(t)*\sigma (t)$$

From that, the respective integrated sensitivities, as a measure of overall parameter importance, are obtained by integrating $\hat{S}(t)$ over the signal length:


(3) $$\hat{S}=\int S_{i}(t)*\sigma (t)dt.$$

The integrated sensitivities are given in [mVs], i.e. measurable signal change in *mV* over the signal length, and best reflect which parameter (combinations) introduce measurable change in the 12-lead ECG signal. By normalizing them, i.e. by dividing through the time-integrated σ=∫σ(t)dt, one obtains relative proportions of parameter importance $S_{i}$. Per definition, the sum of all $S_{i}$ is 1, similar to the Sobol coefficients.


(4) $$S_{i}=\frac{\int S_{i}(t)*\sigma (t)\;dt}{\int \sigma (t)\;dt}.$$

To gauge the surrogate reconstruction error, we reconstructed a random number of ECGs using the PCE with parameters used to generate ECGs in the synthetic ECG database. The $L_{2}$ norm was then computed between each ECG pair for comparison, and the mean and standard deviation were computed for each ventricle. For full details on the sensitivity analysis, please refer to Supplementary material online, *S4*.

### ECG genesis in septal accessory pathways

Three simulations were selected to exemplify the genesis of the 12-lead ECG in right-sided and left-sided septal accessory pathways. The first simulation was the personalized sinus rhythm generated by the patient-specific model of the single subject. Simulations of two APs in the septum were then selected from the 12-lead ECG database. The two septal APs had similar insertion sites within the atria but exit sites on opposed sides of the septum to modulate left-sided and right-sided pathways. Both exit sites were located in close proximity to the existing His–Purkinje system, but not directly connected. The ventricular activation time in both APs was set to be a value of 125ms from the onset of the sino-atrial node activation facilitating a realistic PR interval. The parameters for the two exit sites thus become:


(5) $$\omega_{\mathrm{AP},RV_{\mathrm{septum}}}=\{125\;\mathrm{ms},x_{\mathrm{ven}}(1.0,0.53,-0.49)\}$$

(6) $$\omega_{\mathrm{AP},LV_{\mathrm{septum}}}=\{125\;\mathrm{ms},x_{\mathrm{ven}}(0.0,0.59,-0.45)\}.$$

For all three simulations, the membrane voltages, electrical potentials, and 12-lead ECGs were computed using the simulation framework described in Cardiac simulation section run in pseudo-bidomain mode. A body-surface potential map could be constructed from the electrical potentials. The minimum dV/dt was computed in the downstroke of the S wave for lead V2 for both septal APs.

## Results

An automatic workflow to generate a whole-heart patient-specific model of electrophysiology and incorporate an AP with limited manual user interaction could be constructed from non-invasive clinical data (*Figure* 1). Construction and localization of APs required approximately 1 h. Subsequent generation of the synthetic ECG database of 9,271 signals of left-sided and right-sided APs required under 3d using 16 cores on a desktop machine.

### Regional variation in the 12-lead ECG database

A database was generated by varying AP locations across both ventricles (see *Figure* 2). Sampled sites and their corresponding 12-lead ECGs were organized into regional assignments according to the bullseye plot segmentation (*Figure* 2*B*). Locations of APs evenly cover the atrioventricular junction region on the ventricular side (*Figure* 2*A*) in the upper two apicobasal regions such that the number of regional groupings could be reduced to only 12 within the RV and LV each. The computed $t_{\mathrm{ven}}$ were within the range of 58 to 195ms.

**Figure 3.**
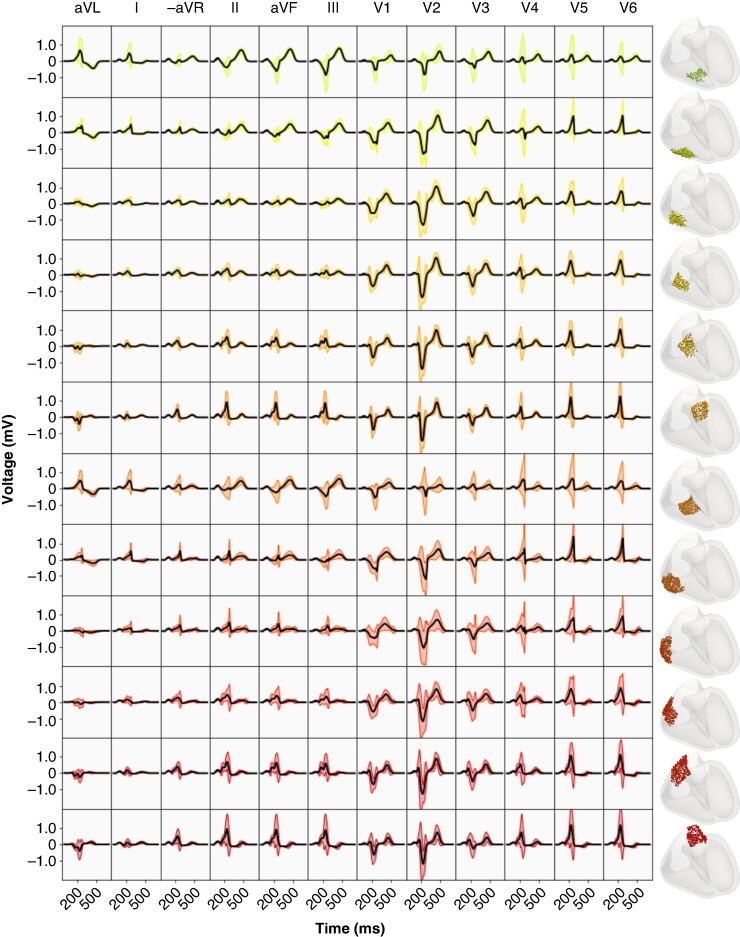
Regional variation in 12-lead ECG morphology stemming from right-sides APs within the 12 regions in the RV (columns). Insertion sites $x_{\mathrm{ven}}$ for each AP are indicated within the corresponding region of the bullseye plot (right). The mean signal is black, and the coloured signals represent the mean ± 2 SD. AP, accessory pathway; ECG, electrocardiogram; RV, right ventricle.

Expected morphological features are present within the 12-lead ECGs for the single subject for both right-sided and left-sided APs. Namely, within right-sided APs (*Figure* 3), the polarity of the V1 lead is predominately negative and within the LV, there is less consistency pertaining to the polarity of the V1 precordial lead. Sites within the anterior free wall of the LV exhibit morphological similarities across multiple rotational regions.

### Sensitivity analysis

The relative errors for the performed PCEs were 16.8%±2.7% for the LV dataset and 16.3%±3.2% for the RV dataset averaged over all leads of the 12-lead ECG. Following Winkler *et al.*^30^, we use the relative error with respect to the signal variance over the whole dataset to quantify the convergence of the PCE surrogate on the chosen parameter interval.^30^

In terms of measurable changes in signal amplitude (*Figure* 5*B,C*), the statistical analysis shows that large measurable changes can be best observed in lead V2 for the LV and the RV datasets due to the high signal variation caused by adding an AP. For the single parameter variations, the ventricular activation time $t_{\mathrm{ven}}$ and the rotational placement ϕ are important, whereas *ρ* and *z* result in no significant signal change. For the second-order variations, the combined contributions of ($t_{\mathrm{ven}},\phi$) and ($t_{\mathrm{ven}},\rho$) give the biggest contributions. More specifically for LV data, the ($t_{\mathrm{ven}},\rho$) induced variations that are largest for V2–V5. The joint contribution of ($t_{\mathrm{ven}},\phi$) induced changes the leads aVL and the inferior leads II, aVF, and III. For the RV data, ($t_{\mathrm{ven}},\rho$) gives the strongest sensitivity for lead V2 and smaller values for V3, V4, and V5. The combined variation of ($t_{\mathrm{ven}},\phi$) causes strong changes in all leads of the RV dataset, except for lead I and -aVR.

Regarding the relative sensitivities $S_{i}$ (*Figure* 5*D*), second-order sensitivities dominate. Within left-sided APs, highest second-order sensitives are seen in leads aVL, aVF, and III stemming from the contribution ($t_{\mathrm{ven}},\phi$). First-order sensitivities are observed in leads -aVR, V2, and V4 to V6 due to variations in excitation time ($t_{\mathrm{ven}}$) in the LV. Within right-sided APs, all leads except V2 to V5 are highly sensitive to the joint contribution of ($t_{\mathrm{ven}},\phi$). Minor second-order sensitivities are observed in leads V4 and II within the LV from the joint-contribution of time and transmural depth ($t_{\mathrm{ven}},\rho$). Some second-order sensitives are also observed in RV for V2 and V3 depending on ($t_{\mathrm{ven}},\rho$). With the exception of -aVR, the rotational placement of the AP influences all limb and Goldberger within the RV.

### ECG genesis in septal accessory pathways

The locations of the right-sided and left-sided septal antegrade APs can be visualized in *Figure* 2*C*. The downstroke of the S wave in V2 is steeper in the right-sided AP with a minimum dV/dt of −0.37 in comparison to −0.23 in the LV septal AP.

**Figure 4.**
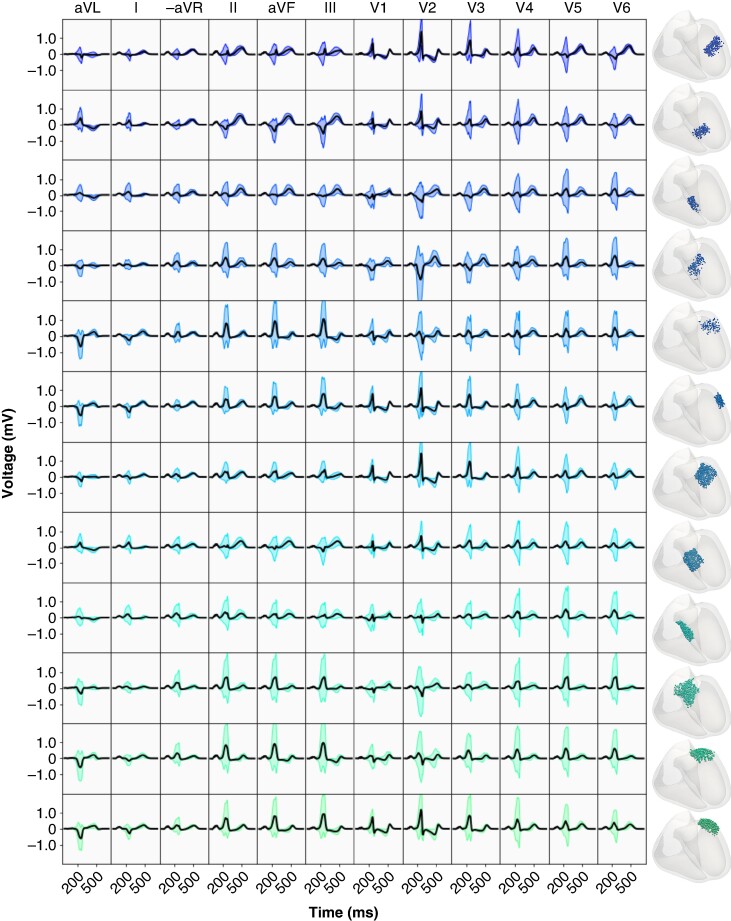
Regional variation in 12-lead ECG morphology resulting from left-sided APs within the 12 regions in the LV (columns). Insertion sites $x_{\mathrm{ven}}$ for each AP are indicated within the corresponding region of the bullseye plot (right). The mean signal is black, and the coloured signals represent the mean plus or minus two standard deviations. AP, accessory pathway; ECG, electrocardiogram; LV, left ventricle.

The 12-lead ECGs generated from septal APs have very similar morphological features (*Figure* 6) that result in both APs being localized to the RV by most existing diagnostic trees. Most importantly, the signals both have a negative (or biphasic) V1 deflection and entirely positive inferior leads (leads II, aVF, and III). Both signals have similar R-wave progression, with the most positive delta wave occurring in lead II. Differences include a longer PR interval in a right-sided AP that can be contributed to the delayed capture of the left bundle of the His–Purkinje System. Noticeable amplitude and morphological differences are observed in leads V1 and V2. The left-sided AP has a weaker S-wave deflection in V1 through V3 and a rSr’ or M shape within V1. The right-sided AP exhibits an rS morphology in V1 and V2, as well as more positive inferior leads. The precordial QRS transition, defined as the occurrence of the R-wave exceeding the S-wave amplitude, occurs in V2 and V3 for the LV and RV sites, respectively. Both sites are located in the fourth regional grouping as displayed in *Figure* 4.

**Figure 5.**
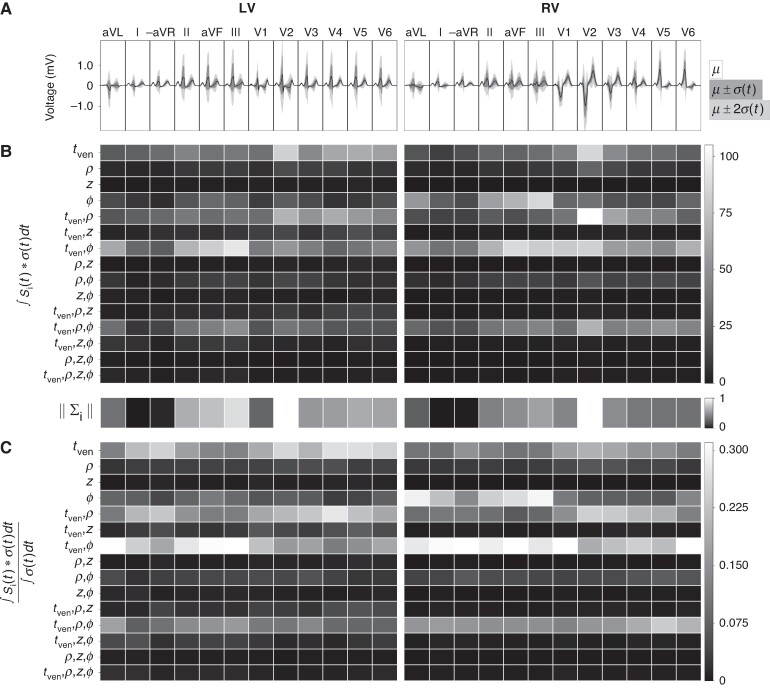
Sensitivity analysis for APs within the RV and LV. (*A*) Mean and standard deviation of the 12-lead ECGs stemming from APs within the LV (left) and RV free wall (right). Time for the ECG is not shown, but lasts 700ms. (*B*) Measure of overall parameter importance in terms of measurable changes in ECG amplitude by integrating $\hat{S}(t)$ over the duration of the signal. The normalized total sum across all sensitivities for each lead is provided underneath. Time-dependent sensitivity $\hat{S}(t)$ of each parameter is not directly shown. (*C*) Relative proportions of parameter importance $S_{i}$. AP, accessory pathway; ECG, electrocardiogram; LV, left ventricle; RV, right ventricle.

**Figure 6.**
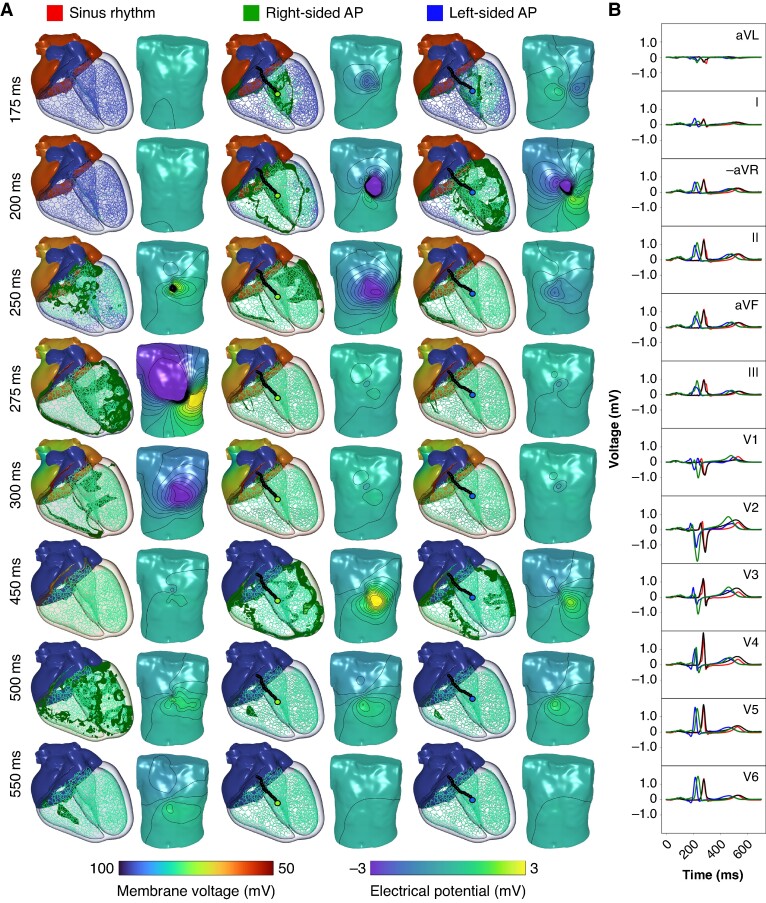
Cardiac activation during normal sinus rhythm (left, red) compared with right-sided AP (center, green) and left-sided APs (right, blue) that are activated at $t_{\mathrm{ven}}=125.0\mathrm{ms}$ and located on opposites sides of the septum at similar apicobasal depth. For all cases, the membrane voltages are shown within the heart and the electrical potentials are displayed on the torso surface at various time points through the cardiac cycle. Locations of the AP can be seen in *Figure* 2*C*. The video representation of this figure is provided in Supplementary material online, *S5*. AP, accessory pathway.

Both common algorithms of Pambrun *et al.*^12^ and El Hamriti *et al.*^11^ were unable to properly locate an AP inserting on the endocardial LV septum within the patient-specific model of WPW as demonstrated through exemplary simulations of two different APs. Within the diagnostic tree from Pambrun *et al.*,^12^ the right-sided septal site would be localized to the appropriate anterior RV septum (NH) due to three positive precordial leads, a negative V1, and a positive V3. The left-sided AP would be localized to the RA for similar morphological reasons in combination with a negative V3. According to Easy-WPW by El Hamriti *et al.*,^11^ both AP sites would be localized to the anteroseptal within the RV. This stems from the negative V1, a precordial QRS transition observed starting in V3, and the most positive delta wave occurring in lead II.

## Discussion

We present a detailed patient-specific model of cardiac electrophysiology that has been adapted to represent an antegrade AP in the form of a short atrioventricular bypass tract in WPW syndrome generated within an efficient workflow. The virtual model was used to provide mechanistic insights into the morphological genesis of the 12-lead ECG for improving both sensitivity and specificity in clinically deployed ECG-based algorithms. Improvements in ECG-based algorithms allow for more accurate non-invasive AP localization to avoid the need for cardiac imaging or electrophysiological studies during the ablation procedure. The efficient workflow for generating a patient-specific model of WPW also has the potential to become a clinically viable approach for personalized care for patients with complex disease representations. Patient-specific models could facilitate tailored ablation strategies, assess the risk of sudden cardiac death, or guide management plans for asymptomatic patients.^3,56^

### Analysis of ECG-based localization algorithms

Performing sensitivity analysis on virtual models provides a novel means to obtain insight into the morphological genesis of the 12-lead ECG within WPW in high fidelity. Signal-derived SA highlights the locations in the 12-lead ECG that depend on parameters relating to the AP placement and timing (Sensitivity analysis section). First, the joint contribution from the rotational placement and activation time of the AP creates the strongest measurable change in the V2 precordial lead for sites located within the RV free wall. Measurable amplitude change in V2 for all APs is also tied to ventricular activation time of the site ($t_{\mathrm{ven}}$). When considering the general placement of V2 in proximity to the heart, delayed activation of the LV free wall in comparison to the RV as seen in right-sided APs leads to a generally larger slurred S wave in V2 (*Figure* 3). This is further exaggerated when the AP is located in the RV free wall. Inversely, a more positive S deflection stems from sites located in the LV free wall (*Figure* 4). Second, both left- and right-sided APs induce larger morphological changes in the inferior leads (II, aVF, and III) in both left- and right-sided APs (*Figure* 5). This stems from the AP-triggered ventricular dyssynchrony that causes a slight shift in the axis of the heart leading to a shift in the QRS transition and R-wave progression. Most standard ECG localization algorithms thus expectantly encode the electrical heart-axis observed in these leads in some way; El Hamriti *et al.*^11^ use the lead with the most positive delta wave and precordial QRS transition and^12^ relies on the number of positive leads.

Regarding the resolution of current ECG algorithms, El Hamriti *et al.*^11^ states that the use of 10 regions is sufficient to differentiate between characteristics within the 12-lead ECG, and the use of further regions complicates clinical decision-making. Our computational modelling supports this. The majority of signal variation (*Figures* 3 and 4) can be observed within less than 10 rotational regions within the LV and RV combined. Additionally, as the insertion depth plays little role in signal morphology as indicated in *Figure* 5, a pure rotational-based regional separation in an ECG algorithm may suffice. Some anatomical regions present in ECG algorithms, such as the deep coronary sinus,^12^ were not accounted for within the patient-specific model of WPW.

As indicated by the example simulations of APs located on either side of the septum (ECG genesis in septal accessory pathways section), differentiating between a bundle branch block pattern in leads V1 and V2, instead of relying on the polarity of the V1 lead alone, may improve differentiation between left- and right-sided APs. Within the right-sided AP, the RV activates slightly prior to the LV even with an intact His–Purkinje system. The right-sided pathway thus exhibited a stronger S-wave deflection within V1 and V2 (*Figure* 6). This corresponds with a slight left bundle branch block pattern. A later precordial QRS transition is also observed in the right-sided site, which is indicative of left bundle branch block. Inversely, the left-sided AP experienced a rS shape in both V1 and V2 stemming from later activation of the RV. These morphological traits are more characteristic of right bundle branch block pattern. Algorithms for localizing idiopathic ventricular tachycardia typically rely on similar criteria and principles.^57^ Further differentiation could be done by looking at the downstroke of the S wave in lead V2, where right-sided APs will have a stronger S wave due to delayed activation of the left ventricle.

Various algorithms based on machine learning also show promise for the purpose of automatic localization,^58,59^ but suffer from similar limitations and lack of sufficient clinical data for training. Supplementing training data for such approaches with synthetically trained 12-lead ECG data may be useful in training algorithms. Our 12-lead ECG database on WPW is the first publicly available database of its kind that could be utilized towards such an end. The synthetic ECG database generated for this study can be found on Zenodo with DOI 10.5281/zenodo.10949804.

Towards the aim of generating even larger synthetic ECG databases for machine learning, the UQ approach relies on constructing a surrogate model that allows for instantaneous simulation. The underlying PCE-based surrogate model in this study, however, is likely not suitable to replace the forward simulations with sufficient fidelity given the high signal-averaged relative errors of 16.8%±2.7% for the LV and 16.3%±3.2% for the RV dataset. Surrogate models generally converge worse with an increasing number of parameters, larger parameter ranges, and substantial signal variance caused by specific parameter changes. These large errors reflect the complex dependency of the ECG on the parameter variations and the possible non-uniqueness of the problem. Performing SA using predetermined ECG-derived metrics such as V1 polarity may result in better approximations, and reduce errors. While the estimated surrogate errors are still sufficient for a meaningful SA, different PCE terms used could lead to slightly different sensitivities.

### Patient-specific models of WPW syndrome

Accurate prediction of patient-specific AP localization using patient-specific models could aid decision-making regarding initiation of medical treatment,^60^ especially in currently asymptomatic patients,^56^ but also regarding indication of an electrophysiological study. Given real patient data, the model could be personalized to the 12-lead ECG of a patient with WPW to quantify the most probable location of an AP to reduce the need for costly electrophysiological studies during an intervention. This would amount to solving a specific ECG inverse problem as seen in electrocardiographic imaging.^60^ Note that the achieved accuracy is adequate for identifying the major parameter sensitivities, however, it is insufficient for employing the surrogate in solving the inverse problem. Challenges in model personalization must first be overcome as later discussed, but recent efforts in this direction have been made.^61,36,62^

Uncertainties in patient-specific modelling arising during both anatomical model generation and calibration may influence simulation predictions as well as clinical outcomes.^63^ In terms of anatomy, the patient-specific model in this work was generated from MRIs that are not standard clinically and changes in imaging modality influence the accuracy of the anatomical model.^64^ The anatomical model generation pipeline has been extended to work on various other imaging data including standard cardiac MRI and computed tomography.^65^ The MRIs were registered together resulting in an error on the order of 1 or 2cm stemming from breathing displacement, segmentation errors, and the registration itself. Calibration errors must also be considered when performing patient-specific modelling and may influence outcomes. Sources include generically calibrated parameter values (as seen in the atria in the patient-specific model), assumptions in the biophysical model that cause deviations from reality, and the non-uniqueness of the inverse problem. Further validation is needed to explore the influence of anatomical model generation and calibration.

Testing the viability of using the patient-specific models of WPW to do pre-procedural planning could be carried out within the context of a clinical study. While the 12-lead ECG results support a valid representation of WPW syndrome, model evaluation on real clinical data has not yet been performed. The current model was parameterized under sinus rhythm and then extended for WPW syndrome. Cardiac imaging in these patients of any imaging modality is however not standard and thus it is difficult to build patient-specific models. Furthermore, clinical datasets consisting of electro-anatomical mapping that are needed for validation are not recorded except under the circumstance of a co-existing condition necessitating additional treatment. Even when invasive procedural data is available, it is typically only available as an electro-anatomical map on a limited endocardial manifold on either side of the atrioventricular junction. To assess model credibility for clinical use, SA may also be useful.^66^

### Translational impact

The computational modelling platform we have developed has significant translational impact. In general, personalized models in the form of cardiac digital twins or patient-specific models that accurately reflect individual patients in terms of both cardiac anatomy and electrophysiology enable highly personalized treatment planning, surgical planning, and risk stratification.^21^ This personalized approach can also be extended to other arrhythmias and structural heart diseases with simple extensions, enhancing the precision and efficacy of interventions. This patient-specific model closely resembles an idiopathic ventricular tachycardia and can be used to help train algorithms for localization of ectopic or focal beats stemming from the ventricles.^67,60^ Extensions to a cardiac solver allowing re-entry would be required for modelling atrial fibrillation and ventricular tachycardia that coincide with WPW. Many patient-specific models have also been developed for assisting in ablation and treatment protocols^68,22^ and these conditions often coincide with WPW.^5^ In patients with heart failure, our patient-specific models could simulate the effects of cardiac re-synchronization therapy and optimize lead placement for improved therapeutic outcomes.^69,70^ Patient-specific models are also particularly valuable for conditions like hypertrophic cardiomyopathy that can occur with familial WPW, where the risk of sudden cardiac death can be assessed more accurately through personalized simulations. By simulating various clinical scenarios, platforms for generating patient-specific models in a cohort can be used for *in silico* clinical trials^62^ and stratifying risk profiles. This can also aid in pre-surgical planning and postoperative management.

Other broader potential benefits span across education and training, research and development, pharmacological studies, and public health and policy. In medical education and training, the platform can serve as an advanced educational tool for medical students, residents, and fellows as it provides a dynamic way to learn about cardiac electrophysiology, arrhythmias, and the impact of various interventions. For electrophysiologists and cardiologists, the platform offers a sophisticated simulation-based training environment. Practitioners can hone their skills in identifying APs, interpreting ECGs, and planning ablation procedures without risk to patients. Towards pharmacological studies, *in silico* models can accelerate drug development and assess potential anti-arrhythmic therapies. The platform can also be extended to study the effects of various pharmacological agents on cardiac electrophysiology in a controlled *in silico* environment.^71^ This would warrant including complex cellular dynamics between drugs and various ion channels.^72^ By aggregating data from multiple patient-specific simulations, our platform can contribute to large-scale epidemiological studies, providing insights into the prevalence and management of various cardiac conditions. The evidence generated through simulations can also inform health policy decisions, particularly in optimizing resource allocation for cardiac care and improving guidelines for arrhythmia management.

### Future work and limitations

All the clinical applications of AP localization prediction become even more important when dealing with paediatric patients. Although most algorithms to predict AP localization were derived from adult patients, the paediatric population carries the highest burden of WPW syndrome with age-related peaks of arrhythmias appearing during infancy and adolescence. Incorporation of paediatric ECG data on a whole-heart model of a paediatric patient suffering from WPW syndrome may allow for more accurate algorithms in the future. This study, however, was performed within a single patient-specific model of a healthy adult male who was equipped with an antegrade AP to represent WPW. The methodology should be extended to a cohort of children and adolescents, the majority of the clinical population, as changes in the heart axis due to age may influence both results and the efficacy of such algorithms in children.^11^ As acquisition of cardiac images in patients with WPW is not clinically standard thus limiting cohort generation, constructing shape-models or atlases of paediatric^73^ or adult^74^ hearts from a limited number of scans may be a viable option for larger population studies on WPW and ECG localization algorithms.These models have been also paired together with SA.^28^

Assessment of how variations in AP conduction and placement impact a clinical ECG diagnostic method is needed. We assumed a maximal AP length of d=6cm of the sites in order to investigate the role of AP placement and excitation across the top portion of the ventricles with no restrictions on atrial placement. In future work, further physiological restrictions in universal cardiac coordinates on both $x_{\mathrm{ven}}$ and $x_{\mathrm{atria}}$ defining the APs can be done to explore specific types of pathways of varying lengths and types.^75,50^ Furthermore, the conduction velocity in the AP ($CV_{AP}$) was prescribed to be fairly fast at $2.0{m\;s}^{1}$ based on reported electrophysiological properties and behaviour of atriofascicular pathways in patients with pre-excitation syndromes and Mahaim fibres.^50^ Conduction may be slower in APs that are located in different areas of the heart.^76^ Variations in conduction velocity, and thus $t_{\mathrm{ven}}$, should therefore be explored. Multiple APs with different conduction velocities could also be considered.^75,19^

While not available at the time of the study, re-entrant simulations would facilitate modelling APs allowing typical atrioventricular conduction, but retrograde propagation through the atrioventricular junction region, as well as simulations of multiple heartbeats. Re-entrant circuits would necessitate accounting for restitution in conduction velocity and action potential duration stemming from rapid cycle lengths. We reported a technique for tuning these properties in previous work.^77^ The patient-specific model could then be used to explore disease mechanisms and morphological changes in the 12-lead ECG when WPW is in conjunction with other cardiac diseases and manifestations such as atrial fibrillation, bundle branch blocks, ventricular tachycardia.^1,5^

The ECG database could be generated in under 3 days, but using simpler models or alternative sampling schematics could decrease these computational costs. Simpler and lighter patient-specific models without full representations of the HPS may be capable of capturing similar morphological elements on the ECG as indicated in previous work^47^ and can thus be useful in understanding basic mechanisms of the disease or even directly for pre-excitation localization as shown in Berger *et al.*^60^ using electrocardiographic imaging.^60^ They may thus be more efficient for population studies without a loss of fidelity. Further investigation into model differences on ECG morphology and underlying mechanisms would be needed. A more informed sampling of APs could also decrease the ECG database generation, as it is known that APs are not distributed uniformly across the atrioventricular plane.^76^ Alternative sampling approaches may be more efficient than latin-hyper-cub sampling, but a speed-up was not observed in similar work using Sobol sampling.^30^ For procedural planning in individual patients, a highly detailed patient-specific model with a full representation of the HPS is warranted especially in the instance of compounding conditions or retrograde APs.

## Supplementary Material

euae223_Supplementary_Data

## Data Availability

The synthetic ECG database of a specific type of WPW syndrome generated within this study can be found on Zenodo with DOI 10.5281/zenodo.10949804. The model and clinical data used within this study are not publicly available. Many tools used to generate the pipeline are available in the openCARP framework^37,49^. All other tools require specific collaboration agreements.
