# Supplementary material for: A computational study on the influence of antegrade accessory pathway location on the 12-lead electrocardiogram in Wolff–Parkinson–White syndrome
Source: Europace. 2024 Sep 11;27(2):euae223. doi: 10.1093/europace/euae223 (PMC11879338; doi:10.1093/europace/euae223)
Supplement: euae223_Supplementary_Data [file euae223_supplementary_data.zip › gillette_2024_wpw.EP-Europace.supplement.submission.pdf]

Supplementary Material

S1 Representation of the Accessory Pathway

A short AV bypass tract allowing antegrade conduction was added into the CDT to replicate ventricular pre-excitation caused by WPW. It was assumed that the insertion site ($\mathbf{x}_{\text{atria}}$) in the atria and the exit site ($\mathbf{x}_{\text{ven}}$) in the ventricles connect on the atrial or ventricular sides, respectively, of the AV junction region at a maximal distance d_{max} apart. To enforce this, sites $\mathbf{x}_{\text{ven}}$ and $\mathbf{x}_{\text{atria}}$ must be within a circle of diameter d_{max} centered at a point on the AV junction region, x_{b} such that:

$$\|\mathbf{x}_{\text{ven}} - \mathbf{x}_{\text{b}}\| \leq \frac{d_{\text{max}}}{2} \qquad \|\mathbf{x}_{\text{atria}} - \mathbf{x}_{\text{b}}\| \leq \frac{d_{\text{max}}}{2} \qquad \|\mathbf{x}_{\text{ven}} - \mathbf{x}_{\text{atria}}\| \leq d_{\text{max}}$$

All points can be defined using anatomically intuitive UAC and UVC positioning where

$$\mathbf{x}_{\text{b}} \in \{D_{\text{UVC,RV}}, D_{\text{UVC,LV}}\}, \quad \mathbf{x}_{\text{atria}} \in D_{\text{UAC}}, \quad \mathbf{x}_{\text{ven}} \in \{D_{\text{UVC,RV}}, D_{\text{UVC,LV}}\}$$

with

$$\begin{aligned} D_{\text{UVC,LV}} &= \{\rho, z, \phi, \nu \quad | \quad \rho \in [0,1], z \in [0,1], \phi \in (-\pi, \pi], \nu = -1\} \\ D_{\text{UVC,RV}} &= \{\rho, z, \phi, \nu \quad | \quad \rho \in [0,1], z \in [0,1], \phi \in (\frac{-\pi}{2}, \frac{\pi}{2}), \nu = 1\} \\ D_{\text{UAC}} &= \{\gamma, \alpha, \beta, \nu \quad | \quad \gamma \in [0,1], \beta \in [0,1], \alpha \in [0,1], \nu \in \{-2, 2\}\} \end{aligned}$$

Exact localization of the sites into nodes existing on the mesh was facilitated through open-source *meshtool* that relies on a custom-built KD-tree algorithm [1].

To connect the two sites and form an AP, a geodesic path was found between the two sites and the length d computed.

$$d = \|\mathbf{x}_{\text{atria}} - \mathbf{x}_{\text{b}}\| \qquad s.t \qquad d \leq d_{max}$$

Within the AP, a conduction velocity of $2.0\,\text{m}\,\text{s}^{-1}$ was set to replicate His-Purkinje fibers, such that the prescribed timing of the pre-excitation (t_{ven}) could be computed as:

$$t_{\text{ven}}(\mathbf{x}_{\text{ven}}) = \frac{d}{2.0} + t_{\text{atria}}(\mathbf{x}_{\text{insert}})$$

S2 Sampling Schematic

A synthetic ECG database was then generated by varying AP locations across both ventricles. The point $\mathbf{x}_{\text{b}}$ on AV junction was restricted to the basal surface ($z = 1.0$) and assumed to lie halfway through the ventricular wall ($\rho = 0.5$) reducing the site to be defined only be the ϕ coordinate in either the LV or the RV. Using Latin hyper cube sampling, 10k locations for $\mathbf{x}_{\text{b}}$ were selected through the entire ϕ range in the respective ventricular domain. A maximum allowed distance of $d_{max} = 6.0\,\text{cm}$ was then allowed between $\mathbf{x}_{\text{ven}}$ and $\mathbf{x}_{\text{atria}}$, and the two sites were randomly selected within their respective chambers within the search radius from any given point $\mathbf{x}_{\text{b}}$. Given this representation of the AP, a parameter vectorω consisting

of a total of 4 parameters defines differences between 12-lead ECGs in either the LV or RV and the pre-excitation that occurs in the ventricles:

$$\omega_{\mathrm{AP,RV}} = \{t_{\mathrm{ven}}, \mathbf{x}_{\mathrm{ven}}(\rho, z, \phi)\} \in D_{\mathrm{UVC,RV}} \tag{S1}$$

$$\omega_{\mathrm{AP,LV}} = \{t_{\mathrm{ven}}, \mathbf{x}_{\mathrm{ven}}(\rho, z, \phi)\} \in D_{\mathrm{UVC,LV}} \tag{S2}$$

Note that sites defined in $D_{\mathrm{UVC,LV}}$ with $\rho > 0.5$ and $-\pi/2 < \phi < \pi/2$ may actually be denoted as clinically right-sided since the APs are anchoring in the RV septum. The RV outflow tract was taken to be inert and thus $\mathbf{x}_{\mathrm{atria}}$ were neglected within this area. A final total of 4678 APs in the LV and 4593 in the RV were actually sampled across the AV junction region. The computed t_{ven} were within the range of 58.0 to $194.0\,\mathrm{ms}$.

S3 Bullseye Plot Construction for Regional Separation

The bullseye plot was constructed with UVC-based cutoffs. Four apico-basal cutoffs in both ventricles were applied such that $z = \{0.2, 0.4, 0.7\}$. The top two apico-basal regions were partitioned into 6 rotational regions with cutoffs at $\phi = \{-2.2, -1.1, 0, 1.1, 2.2\}$ within the LV and $\phi = \{-1.0, 0.5, 0, 0.5, 1.0\}$ within the RV. The third apical-basal region with $z = [0.2, 0.4)$ was assumed to have only four regions within the LV such that cutoffs were applied at $\phi = \{-1.47, 0.21, 1.89\}$. Within the RV, 3 rotational regions were assigned in the third apico-basal region with cutoffs $\phi = \{-0.5, 0.5\}$.

S4 Sensitivity Analysis

Quantifying the sensitivity of the 12-lead ECGs from the database with respect to the four different input parameters for the RV and LV, $\omega_{\mathrm{AP,RV}}$ Eq. (S1) and $\omega_{\mathrm{AP,LV}}$ Eq. (S2), was performed by employing PCE [2]. Thereby, the signal variance, obtained from model evaluations with given input parameter values, is decomposed into parts resulting from single parameter variations and simultaneous variations of multiple input parameters via Sobol indices (SI) [3]. In PCE, one obtains an approximation of the forward model on the sampled parameter space that directly enables the calculation of all SI further including upper bounds for the respective errors. This results in a so-called *global sensitivity analysis* relaying parameter dependencies over the whole sampling range - in contrast to local approaches based on gradient estimates, cf. [4] for a more comprehensive treatment of the procedure, developed for atrial ECG data. With the surrogate model, it becomes further possible to quickly sample more, approximate model evaluations, facilitating, for e.g., the quantification of specific parameter uncertainties.

We use here a non-intrusive approach working on arbitrarily sampled model evaluations of uniformly distributed input parameters. It explicitly enables gradual sampling refinement until the surrogate accuracy is deemed sufficient. More specifically, we applied the Python toolbox “PyThia UQ” that determines PCE expansion coefficients via a multi-linear least-squares regression [5]. Given a sampled dataset of model evaluations, the best fitting PCE model is obtained by optimizing the hyperparameters governing the distribution of PCE terms with respect to the resulting surrogate error, cf. equation S3.

In the present case, the PCE was performed by considering terms up to the 6th order for the RV, and up to the 7th order for the LV dataset, which yielded the lowest surrogate errors. To gauge the surrogate error, we calculated the L_2 norm of a randomly chosen test dataset, where known ECG-parameter pairs (ground truth, GT) were compared to curves reconstructed by the PCE:

$$E_2 = \sqrt{\int [GT(t) - PCE(t)]^2 \, dt} \tag{S3}$$

E_2 averaged over the test dataset gives an indication of the PCE convergence and reliability of the SA derived by calculating the PCE-based Sobol indices. Relating E_2 to the total standard deviation of the signal in a given dataset encoded the amount of information gained by constructing the PCE in a statistical sense.

S5 Video of Example Simulations of Septal Accessory Pathways

This video titled 'S5_fig6_video_representation.final.mp4' corresponds to the visualization of Fig. 6 in the main manuscript. Videos of both membrane voltages on the heart and electrical potentials on the torso surface are shown for normal sinus rhythm, an AP in the LV septum, and an AP in the RV septum. The order of the video corresponds to the same order in Fig. 6.

REFERENCES

[1]Aurel Neic, Matthias AF Gsell, Elias Karabelas, Anton J Prassl, and Gernot Plank. Automating image-based mesh generation and manipulation tasks in cardiac modeling workflows using meshtool. *SoftwareX*, 11:100454, 2020.

[2]Bruno Sudret. Global sensitivity analysis using polynomial chaos expansions. *Reliability engineering & system safety*, 93(7):964–979, 2008.

[3]Ilya M Sobol. Global sensitivity indices for nonlinear mathematical models and their monte carlo estimates. *Mathematics and computers in simulation*, 55(1-3):271–280, 2001.

[4]Benjamin Winkler, Claudia Nagel, Nando Farchmin, Sebastian Heidenreich, Axel Loewe, Olaf Dössel, and Markus Bär. Global sensitivity analysis and uncertainty quantification for simulated atrial electrocardiograms. *Metrology*, 3(1):1–28, 2022.

[5]Nando Hegemann and Sebastian Heidenreich. Pythia: A python package for uncertainty quantification based on non-intrusive polynomial chaos expansions. *Journal of Open Source Software*, 8(89):5489, 2023.
